# Equitable differential privacy

**DOI:** 10.3389/fdata.2024.1420344

**Published:** 2024-08-16

**Authors:** Vasundhara Kaul, Tamalika Mukherjee

**Affiliations:** ^1^Department of Sociology, Purdue University, West Lafayette, IN, United States; ^2^Industrial Engineering and Operations Research, Columbia University, New York, NY, United States

**Keywords:** differential privacy (DP), Census 2020, inclusive communication, data privacy, equity

## Abstract

Differential privacy (DP) has been in the public spotlight since the announcement of its use in the 2020 U.S. Census. While DP algorithms have substantially improved the confidentiality protections provided to Census respondents, concerns have been raised about the accuracy of the DP-protected Census data. The extent to which the use of DP distorts the ability to draw inferences that drive policy about small-populations, especially marginalized communities, has been of particular concern to researchers and policy makers. After all, inaccurate information about marginalized populations can often engender policies that exacerbate rather than ameliorate social inequities. Consequently, computer science experts have focused on developing mechanisms that help achieve equitable privacy, i.e., mechanisms that mitigate the data distortions introduced by privacy protections to ensure equitable outcomes and benefits for all groups, particularly marginalized groups. Our paper extends the conversation on equitable privacy by highlighting the importance of inclusive communication in ensuring equitable outcomes for all social groups through all the stages of deploying a differentially private system. We conceptualize Equitable DP as the design, communication, and implementation of DP algorithms that ensure equitable outcomes. Thus, in addition to adopting computer scientists' recommendations of incorporating equity parameters within DP algorithms, we suggest that it is critical for an organization to also facilitate inclusive communication throughout the design, development, and implementation stages of a DP algorithm to ensure it has an equitable impact on social groups and does not hinder the redressal of social inequities. To demonstrate the importance of communication for Equitable DP, we undertake a case study of the process through which DP was adopted as the newest disclosure avoidance system for the 2020 U.S. Census. Drawing on the Inclusive Science Communication (ISC) framework, we examine the extent to which the Census Bureau's communication strategies encouraged engagement across the diverse groups of users that employ the decennial Census data for research and policy making. Our analysis provides lessons that can be used by other government organizations interested in incorporating the Equitable DP approach in their data collection practices.

## 1 Introduction

Today, a wide range of personal and sensitive data is regularly collected from individuals by numerous institutional actors such as governments, business organizations, and research institutions. This data is used for various purposes, from making policy decisions and conducting research studies to selling targeted advertisements. Given the far-reaching scope of this data collection, there has been a growing concern surrounding the protections for respondent privacy and anonymity enforced by the data collectors. Of particular concern has been the harm that can arise for individuals if their sensitive data is disclosed improperly. Historically, different methods to anonymize data have been used to protect the privacy of individuals sharing their information. However, computer scientists have consistently shown how even anonymized data is vulnerable to privacy attacks (Sweeney, 2000; Narayanan and Shmatikov, 2008; Ohm, 2010) and that anonymization methods typically do not provide a mathematical measure of privacy loss. Since some amount of privacy loss is inevitable for any publicly available dataset (Dinur and Nissim, 2003), understanding the amount of privacy loss incurred is crucial in effectively protecting individual privacy. As a result, a more formal privacy framework with provable mathematical guarantees on the amount of privacy loss known as differential privacy (DP) was developed (Dwork et al., 2016).

While DP has been used extensively by several large corporations like Google and Apple since 2014 (Erlingsson et al., 2014; Apple, 2017), it became a matter of national concern after the U.S. Census Bureau (henceforth referred to as the Census Bureau or the Bureau) adopted DP for the 2020 Census. Of particular concern has been the decline in the accuracy of datasets as the amount of privacy protection guaranteed by a differentially private algorithm increases (i.e., the privacy-accuracy trade-off) and the subsequent impact of this accuracy loss on marginalized groups (Santos-Lozada et al., 2020; Hauer and Santos-Lozada, 2021).^1^ For e.g., under the Census Bureau's proposed use of DP, population counts will be released after the addition of a controlled amount of noise, i.e., random numerical values specified by DP parameters. Researchers have identified that smaller (marginalized) sub-population counts may be completely skewed by the addition of these random numerical values.^2^ Such distortions are particularly unsettling because an erasure or misrepresentation of sub-populations counts of marginalized communities can have wide-ranging adverse consequences for them and possibly exacerbate existing social inequities.

Concerns of this nature have motivated discussions around the fairness of DP algorithms and how inequities that may arise through distortions introduced by DP computations can be addressed. Specifically, discussions on fair DP algorithms have revolved around designing algorithms where fairness is either quantified using mathematical equations (Xu et al., 2019; Tran et al., 2021) or achieved through the introduction of post processing measures that mitigate some inequities (Pujol and Machanavajjhala, 2021; Steed et al., 2022). In our paper, we extend the conversation on the fairness and equity of DP algorithms by identifying inclusive communication as an essential but fleetingly discussed dimension of implementing Equitable Differential Privacy. While fairness and equity are often used interchangeably in the discussions of fair DP algorithms, we specifically focus on equity since the concept accounts for and addresses the social hierarchies that disadvantage marginalized communities.

First, we formally define *Equitable Differential Privacy* as the design, communication, and implementation of DP algorithms that ensure equitable outcomes. We argue that algorithmic fixes to address inequities by accounting for fairness measures or adding post processing steps to mitigate harms constitute a necessary but insufficient means of ensuring that DP algorithms do not exacerbate social inequities. Therefore, we emphasize the pivotal and complementary role of inclusive communication in achieving this goal. Second, we draw on the inclusive science communication (ISC) framework to identify best practices for communication with non-expert groups to facilitate their inclusion in the development and use of DP systems. Lastly, we undertake a case study on the adoption process of DP as the privacy-preserving mechanism for Census 2020. Given the diverse purposes for which Census data is used, the case study permits us to demonstrate the importance of inclusive communication and knowledge sharing with DP non-experts. We evaluate the extent to which the communication strategies employed by the Census Bureau fall within the ISC framework. Additionally, we identify areas where more work needs to be done to intentionally engage the diverse users of the decennial Census data and address their equity concerns. We conclude our paper by proposing lessons that other U.S. government organizations can learn from the experiences of the Census Bureau about implementing inclusive communication practices that constitute Equitable DP. Our aim is not to provide prescriptive guidelines applicable for all types of organizations on how to attain Equitable DP. Instead, we hope our work will encourage future researchers to undertake similar evaluations of other types of organizations so that we can cumulatively arrive at a general principle of inclusive communication for Equitable DP.

## 2 Understanding equity in the context of differential privacy

### 2.1 What is differential privacy?

Differential Privacy (DP) (Dwork et al., 2016) is a mathematically rigorous privacy-preserving framework that provides quantifiable protection against a wide range of privacy attacks. DP is primarily studied in the context of the collection, analysis, and release of aggregate statistics from databases with records of individuals. A differentially private algorithm takes a database as input and mathematically guarantees that the presence or absence of an individual's record does not drastically change the result of a statistical computation through the addition of some controlled amount of noise. The strength of the privacy guarantee is controlled by the privacy parameter, also referred to as the privacy loss or privacy budget. The smaller the value of the privacy parameter, the more protected each individual's data is, which leads to a greater degradation in the utility of the computation - an exchange often referred to as the privacy-accuracy trade-off. For a more detailed overview of DP literature, see Appendix 1.

In recent DP literature, there has been a push to offer end users more fine-grained explanations of the mathematical parameters used in DP to control privacy loss (Nanayakkara et al., 2022, 2023; Weiss et al., 2023) to avoid privacy theater, i.e., instances where technologies offer the “feeling of improved privacy while doing little or nothing to actually improve privacy” (Smart et al., 2022). Computer scientists have focused on examining how people's (non-experts and users) understanding of DP varies based on type of communication strategy employed (Bullek et al., 2017; Xiong et al., 2020; Cummings et al., 2021; Karegar et al., 2022). But, there has been limited examination of how audience perceptions and needs are included in DP workflows. To address this gap in the literature, we evaluate how diverse audiences are engaged in conversations about DP and the extent to which their ideas are meaningfully incorporated in the design and implementation of Equitable DP. We argue that it is critical to understand the inclusivity in the communication of these design and implementation processes because they directly affect the extent to which DP algorithms obscure the prevalence of or impede the study of social inequities.

### 2.2 What is equitable DP?

Before delving into our conceptualization of Equitable DP, it is first important to understand the meaning of equity. The concept of equity, equality and fairness are closely intertwined but have critical differences pertinent to our conceptualization. Equality refers to the practice of treating everyone the same, regardless of their circumstances or needs. In contrast, equity recognizes that not everyone starts from the same place and circumstances. Thus, equity is ensured through the distribution of resources and opportunities that account for these differences and rectify historical and systemic imbalances so that everyone can arrive at an equal outcome. For instance, a public policy focused on equality treats everyone the same whereas an equitable public policy recognizes that people need different forms of help from the government to arrive at the same outcome. In other words, equality entails formal equality of opportunity, while equity involves substantive equality of opportunity leading to equality of outcome. Fairness is a broader concept that encompasses both equality and equity. The emphasis of fairness is ensuring that processes, systems, and decisions are just and impartial. These goals can be achieved either by either treating people equally when their circumstances are equal (equality) or treating them differently when their circumstances are different (equity).

In the case of algorithms, the concept of fairness is used to understand the justice implications of automated decision-making. Algorithmic fairness is usually quantified through mathematical measures, and currently, there exist many fairness definitions that are applied variably depending on the context of the problem being considered (Kamiran and Calders, 2009; Dwork et al., 2012; Feldman et al., 2015). Fairness in the context of DP has also been an area of growing interest, especially for computer scientists. Bagdasaryan et al. (2019) provide evidence that smaller underrepresented groups are more adversely affected when training DP machine learning models. Similarly, Chang and Shokri (2021) demonstrate how fair machine learning models generate more privacy risks for the underrepresented groups they are trying to protect from algorithmic bias. Cummings et al. (2019) theoretically show that the trade-off between DP and fairness is somewhat unavoidable by proving that a machine learning model cannot be differentially private and exactly satisfy certain fairness constraints. However, they do find that it is possible to construct machine learning models that simultaneously satisfy a weaker notion of privacy (called approximate differential privacy) and several fairness constraints (Cummings et al., 2019; Jagielski et al., 2019; Tran et al., 2021).

These discussions on algorithmic decision-making are not limited to fairness alone. The concept of equity has also been discussed in association with privacy measures in general and DP specifically. Ekstrand et al. (2018) define “fair privacy” as when “the probability of failure and expected risk are statistically independent of the subject's membership in a protected class”. While they do not explicitly define “equitable privacy,” Ekstrand et al. (2018) use the term fairness to reference equitable treatment across classes of people. They develop an agenda for equitable privacy by proposing a list of eleven thought-provoking questions for privacy practitioners and researchers. These questions are centered on assessing the fairness of a privacy protection system (e.g., does the system provide privacy protections to different subject groups?) and, conversely, the privacy of a fairness enhancement scheme (e.g., does the fairness scheme diminish the privacy of its subjects?). In contrast, Pujol and Machanavajjhala (2021) differentiate between the terms fairness and equity. They define *equity* as “equal treatment across groups” whereas *fairness* can encompass several notions of fair treatment of individuals and groups. Their primary focus is to study whether “the disproportionate effects of privacy protections on minority groups result in their unequal treatment in data-driven decision-making.” They examine the trade-off between privacy and equity by studying specific cases of machine learning and allocation problems that use DP to preserve privacy. Through these case studies, they highlight how inequities are introduced in the case of allocation tasks by treating the DP counts of populations as true counts whereas, in the case of machine learning, adding noise to different size groups may cause minority groups to be erased. They discuss several strategies to mitigate the equity issue, such as downstream repair mechanisms.

More recently, Bowen and Snoke (2023) have proposed a guide for defining equity in the context of statistical data privacy (SDP).^3^ They identify two main sources of inequalities that may lead to inequity in the context of SDP: (1) different groups receiving different levels of privacy protection, and (2) different groups accruing disparate levels of social benefit. They demonstrate how different groups can experience different privacy loss and utility by developing a privacy-utility curve for a fictitious dataset from a hypothetical example generated to outline the identified inequalities. Based on this privacy-utility curve, Bowen and Snoke (2023) define equity as “two or more groups being able to place themselves in an equally satisfactory position on the privacy-utility curve”. They emphasize the importance of including equity in all future SDP research to determine how equity can be best balanced with the more traditional inquiries about privacy vs. utility trade-offs.

Overall, we find that fairness and equity are often used interchangeably in discussions about algorithmic decision-making and data privacy. However, since fairness entails both equality and equity, we focus on the concept of equity in our paper. We specifically choose *equity* because of its recognition of how different groups have different requirements to arrive at the same outcome. In the case of DP, this framework allows us to focus on the privacy needs of marginalized and small populations that vary from those of larger populations to ensure that the data of all groups is afforded the same extent of privacy. Thus, we conceptualize “Equitable Differential Privacy” as the design, communication, and implementation of DP algorithms that ensure equitable outcomes for all social groups, particularly marginalized groups. While Ekstrand et al. (2018) and Pujol and Machanavajjhala (2021) mainly focus on the design and implementation of equitable privacy-preserving mechanisms, our definition encompasses design and implementation as well as the *communication processes* employed across all stages. Bowen and Snoke (2023) also discuss the importance of communication at every stage of the data life cycle and the inclusion of different domain experts in the equity conversation. However, they do not provide any recommendations on how this kind of inclusion can be facilitated. In this paper, we emphasize the importance of examining the extent to which communication is established with diverse stakeholders involved in different stages of the DP life cycle, and the mechanisms through which this communication is effectively established. We use the adoption of DP by the U.S. Census Bureau as a case study to evaluate the inclusivity of their communication strategies and the equity implications of the use or absence of such strategies. In particular, we highlight how accessible and engaged communication about DP systems with non-expert audiences has enabled the continued use of the decennial Census data to study social phenomena effectively. Overall, our work highlights the importance of establishing equitable communication and engagement with different domain experts in designing and implementing privacy-preserving mechanisms based on DP algorithms.

## 3 The importance of communication

In a world characterized by rapid scientific and technological developments, the ability to communicate with diverse audiences has become critical for facilitating informed decision-making on these issues. The field of science communication provides several best practice frameworks on how scientific knowledge can be effectively communicated to the general public, especially to combat disinformation and misinformation. However, science communication is often found to trigger the Matthew Effect wherein people engaging in science communication “primarily engage those who seek engagement on our terms, on our turfs, in our language, and in ways that we ourselves find appealing or salient” (Bevan et al., 2020, p. 1). As a result, scientific knowledge often only reaches limited parts of society due to a variety of individual, social, and structural factors (Humm and Schrögel, 2020). In addition, the disseminated knowledge often excludes perspectives held by diverse audiences, thus adversely impacting various outcomes, ranging from individual science career choices (Blanton and Ikizer, 2019) to informed public support for science (Thomas and Durant, 1987). To address these shortcomings, members of the science communication community have been working on developing ways to promote diversity and inclusiveness in science communication.

Science communication frameworks are often based on the deficit model, which presumes that public audiences lack relevant knowledge or experience to understand scientific information (Trench, 2008; Nisbet and Scheufele, 2009; Simis et al., 2016; Smallman, 2016). Typically, the focus of these frameworks is to address the knowledge deficit amongst the broader public by either increasing access to existing pathways to scientific knowledge or increasing the number of pathways. Frameworks based on the deficit model, thus, assume that more points of access will generate increased opportunities for more diverse and representative populations to engage with scientific knowledge and that these populations will choose to pursue these opportunities. However, they fail to account for disparities in the dissemination process of scientific knowledge or the knowledge that the public retains. Increasing access alone does not address other individual, social, and structural barriers that prevent historically marginalized communities' engagement with scientific knowledge. In addition, increasing access does not ensure that marginalized communities' knowledge will be incorporated into the more dominant process of scientific knowledge production. Hence, without a commitment from the scientific community to intentionally engage with socially, racially, and economically diverse communities in science communication, exclusionary systems continue to shape scientific knowledge production and engagement.

### 3.1 Inclusive science communication

In response to these inequities, there has been growing support for the use of an *inclusive science communication (ISC) framework* for the purpose of disseminating scientific knowledge. The framework employs an asset-based approach that assigns value and importance to the ideas, experiences, questions, and criticisms that diverse publics bring to conversations about STEM (Banks et al., 2007). It emphasizes the importance of lay expertise and multiple ways of knowing, and encourages co-creation and collaboration through public participation in science. Therefore, intentional engagement with diverse non-expert groups is central to the idea of inclusive science communication. ISC is characterized by three key traits that exist concurrently: (a) Intentionality, (b) Reciprocity, and (c) Reflexivity (Canfield and Menezes, 2020). These traits are linked by a common thread of equitable relationships and only one trait alone is considered to be insufficient.

1. **Intentionality**. Defined as the “intentional consideration of the audience with whom one is communicating, how science is defined in one's work, and how marginalized identities are, and have been, represented and supported in engagement activities and communication products” (Canfield and Menezes, 2020, p. 14). An approach is considered to be intentional if it promotes collaboration and co-creation with all audiences at all stages, i.e., there is a multi-directional, dialogue-based model of engagement. Intentionality is also achieved when project goals encompass audience and/or community goals, and the cultural histories and backgrounds of the audience are taken into consideration.
2. **Reciprocity**. Building on the idea of intentionality, reciprocity is characterized by the presence of “equitable relationships that recognize and value varied forms of expertise, apply asset-based approaches, and ensure co-created benefit for audiences and communicators/researchers/practitioners” (Canfield and Menezes, 2020, p. 15). The foundational tenet of reciprocity is recognition of the varied expertise of people with different educational backgrounds and lived experiences. Thus, all individuals involved in a project (scientists, practitioners, and community members) are considered to be equally valuable partners.
3. **Reflexivity**. The third ISC trait of reflexivity entails a “continuous, critical, and systematic reflection on the communicators' and audience's personal identities, practices, and outcomes, followed by adaptation as needed to redress inequitable interactions” (Canfield and Menezes, 2020, p. 17). This form of self-reflection can take place on the individual, programmatic, or institutional level. Irrespective of the level, the process calls for consistent self-reflection to assess the implicit biases embedded in the practices being followed by the individual/group/institution, and if the practices are truly able to provide meaningful representation and engagement for marginalized voices.

The use of *boundary objects*, i.e. any object that can be used to facilitate communication across different social groups, is also an approach promoted by the ISC framework to broaden participation in science (Star and Griesemer, 1989; Bevan et al., 2020). A unique feature of boundary objects is that while all stakeholder groups are familiar with the boundary object, each group typically assigns a different purpose, value, and/or meaning to the object (Akkerman and Bakker, 2011). For instance, in their seminal work on boundary objects, (Star and Griesemer, 1989) demonstrate how the shared goal of nature preservation in California, and the creation of standardized processes of information collection and documentation operated as boundary objects that allowed amateur collectors, scientists, and administrative professionals to work together to establish the Berkeley Museum for Vertebrate Zoology. Thus, a key characteristic of boundary objects is their cultural resonance across different local contexts which can be used to develop a shared identity. In fact,“boundary objects not only bridge understanding across people from different positions and locations, they also challenge boundaries, expanding upon who belongs, how and why” (Bevan et al., 2020, p. 3). As a result, boundary objects not only facilitate the inclusion of diverse publics into a scientific conversation but also acknowledge multiple ways of knowing and encourage co-creation of knowledge; features that are essential for inclusive science communication.

### 3.2 Why is communication about DP important?

Since the development and deployment of differential private systems requires niche scientific knowledge that is only possessed by a few, we largely rely on these DP experts to account for equity considerations through fairness measures or post processing harm mitigation. However, in this paper, we propose that the sole use of these equity ensuring algorithmic fixes is based on the implicit assumption that non-experts do not possess the technical knowledge required to ensure equity in the DP development and implementation process. Furthermore, we suggest that it is this biased assumption which limits the involvement of communities that are adversely affected by DP induced data distortions in conversations around Equitable DP, and leads to an over reliance on one form of scientific knowledge. In our paper, we challenge this assumption and submit that the inclusion of non-expert DP audiences brings to fore different equity considerations that are equally important as those addressed by algorithmic fixes. Consequently, the communication strategies used by DP experts to communicate about their systems to non-expert DP audiences, engage and solicit feedback from these audiences, and incorporate feedback from them becomes an integral component of ensuring equity in the the design and implementation of DP systems.

Specifically, we propose that examining the different stages of deploying a DP system through the features of the ISC framework—intentionality, reciprocity, reflexivity, and the presence of boundary objects—allows us (and future researchers) to demonstrate how inclusive communication can help achieve Equitable DP, i.e., ensure that the adoption of DP has an equitable impact on all social groups and does not exacerbate existing social inequities. In particular, since equitable relationships are the underlying factors tying these four ISC features together, we suggest that their presence in the design, communication, and implementation of a DP system reflects that equity has been given ample consideration by the producers of DP systems and that there is a conscious effort to not deepen any existing social inequities. After all, adherence to the ISC framework explicitly calls for intentional engagement with diverse forms of knowledge, particularly from marginalized communities, and their incorporation into mainstream forms of knowledge production and dissemination.

## 4 Methodology

A commonly employed definition of a case study is the “intensive study of a single case or a small number of cases that promises to shed light on a larger population of cases” (Gerring and Cojocaru, 2016, p. 394). The types of case studies can be broadly categorized based on their goals - for causal inference (henceforth referred to as “causal case study”) or descriptive (Gerring and Cojocaru, 2016). As the name suggests, the goal of a causal case study is to demonstrate how a change in X results in a change in Y. While causal case studies do not provide statistically estimated causal effects with confidence intervals, their emphasis is still on determining the effect of X on Y. In contrast, descriptive case studies are primarily concerned with describing a phenomenon. In our paper, we undertake a descriptive case study of the use of differential privacy in the 2020 U.S. Census. We engage in a detailed examination of how knowledge about differential privacy was communicated by the Census Bureau and their differential privacy experts (henceforth collectively referred to as the Census Bureau or the Bureau) to the users of Census data, i.e., academic researchers, policymakers, and advocacy organizations that use Census data for different purposes.

All case studies typically call for a minimum of one or two cases, and the the method by which cases are chosen for detailed examination is referred to as case selection (Gerring and Cojocaru, 2016). Descriptive studies can either employ a “typical” or “diverse” approach to case selection. In a “typical” approach to case selection, a case is selected to reflect select key characteristics of the larger group/population from which it is drawn. For instance, a rural town with a small population can be selected for a case study because it is considered to be typical of other rural towns in the same state with similar populations. However,a case study that employs a “diverse” approach to case selection usually selects a small sample of different cases across a given set of parameters. For instance, a case study of American gun laws could examine one state each which represents a different law toward firearm possession. Using the “typical” case selection approach, we specifically select the Census Bureau for our case study because it is the leading U.S. governmental agency engaged in large-scale data collection at the national level. We propose that the insights gained from the in-depth examination of the Bureau's adoption of DP will be reflective of how other governmental data collection agencies in the U.S. would potentially choose to adopt DP in the future.

Since we are particularly interested in highlighting the role of communication in the establishment of Equitable DP, we examine the Bureau's DP related communication strategies through the ISC framework. We employ secondary sources (e.g., articles in peer-reviewed journals, informational material published by the Bureau, etc.) to determine how users of the decennial Census data - individuals and organizations - were engaged in the process of finalizing the algorithm that implemented DP to the 2020 U.S. Census. We draw attention to processes that encouraged inclusivity by seeking feedback from diverse users and the extent to which the Bureau acted on this feedback. Also, we highlight situations in which the Bureau could have done more to ensure greater inclusiveness at different stages of the process.

## 5 Case study: differential privacy and the U.S. Census

For a background on the disclosure avoidance systems (DAS) used by the Census Bureau prior to adopting the DP-based DAS for the 2020 Census, factors that motivated the transition, and an explanation of demonstration products, refer to Appendix 1.

### 5.1 Multi-year communication and engagement [2016–2021]

Conversations within the Bureau about using a new DP-based DAS began in 2016. After two years of internal discussions, in 2018, the Bureau conducted a simulated reconstruction attack on the 2010 Census data. In this reconstruction attack, the Bureau was able to exactly re-identify 46.48 percent of the population across the five selected variables of sex, age, race, ethnicity and census block. The extent of re-identification even increases to 70.98 percent if age is allowed to vary by +/-1 year (Abowd et al., 2023; Nanayakkara and Hullman, 2023). Based on these findings, the Bureau concluded that their existing confidentiality measures risked disclosing individual responses and began considering moving to a DP-based DAS. However, they were also concerned about how the new DAS could potentially have dissimilar ramifications for different uses of the Census data. Thus, the Bureau requested for feedback from their diverse user community by issuing a Federal Register Notice in July 2018, “Soliciting Feedback from Users on 2020 Census Data Products” (Hotz and Salvo, 2022). Users were asked to provide them with information about which aggregated tables they require (from the ones proposed for the 2020 Census) for their work, “the legal, statutory, and programmatic uses of each data item, along with a request regarding the amount of funding that was distributed based on the data and the level of geography required for the items” (Hotz and Salvo, 2022, p. 10).

Six months later, in December 2018, the Census Bureau released Version 4.0 of their 2020 Census Operational Plan which included information about the DP-based DAS being developed. The release of the Plan was followed by a more public announcement in February 2019 providing the rationale for the Bureau's decision to implement DP in their proposed DAS for the 2020 Census. The announcement included information about their internal re-identification study and how DP helps the Bureau adhere to their duty of confidentiality. In June 2019, the Census Bureau “unveiled” its DP-based DAS called the TopDown Algorithm (TDA) at the Committee on National Statistics (CNSTAT) workshop held in Washington DC and the first demonstration files were issued in October 2019. Hotz and Salvo (2022) refer to this period as the start of the Bureau's 2-year long engagement with the scientific community and the Census data user community to determine whether the TDA has an adverse impact on any of the “use cases” of the Census data.

In December 2019, the Bureau commissioned the CNSTAT of the National Academies of Sciences, Engineering, and Medicine to host a 2-day workshop with the two fold purpose to:“(1) assess the utility of the tabulations in the 2010 Demonstration Product for specific use cases/real-life data applications and (2) generate constructive feedback for the Census Bureau that would be useful in setting the ultimate privacy loss budget and on the allocation of shares of that budget over the broad array of possible tables and geographic levels” (Hotz and Salvo, 2022, p. 12). This workshop brought together a diverse group of researchers who analyzed past Census data and the demonstration data, and presented evidence on the potential impact of the TDA on the different use cases of the Census data. The discussions from these sessions brought to fore critical issues pertaining to privacy-accuracy trade-off in DP such as the lack of usability of the demonstration data for small geographic areas and inaccurate counts for minority communities (National Academies of Sciences and Medicine, 2020). After the December 2019 workshop, academic researchers were not alone in voicing such concerns. Given the substantive equity implications, advocacy organizations were quick to draw attention to the possible harms that could be generated by the Census Bureau's new DAS. Of particular significance to these organizations were the biases that the then current version of the TDA introduced into the Census data which led to inaccurate counts for minority communities. Under-representation of this nature was very troubling because it had a bearing on these communities' political representation, reapportionment, federal funding formulas, accurate research, and local government planning and service delivery (Native American Caucus, 2020; Ochoa and Minnis, 2021).

To address the growing discontent and concerns about the accuracy of the 2020 Census data, the Bureau acknowledged the need to modify the TDA used in the 2010 demonstration products to “optimize the balance between confidentiality and accuracy” (Hotz and Salvo, 2022, p. 14). They identified two sources of error in the TDA—(1) measurement error arising due to the noise added by DP, and (2) post-processing error due to the creation of non-negative integer counts for the purpose of statistical inference (Devine et al., 2020). In March 2020, the Bureau published a set of metrics to allow the public to track the accuracy improvements that would be made to the TDA for different use cases identified by users (Devine et al., 2020). However, many in the data user community found this to be a necessary but insufficient approach, and responded by requesting the Census Bureau to provide new demonstration data files similar to the ones released in October 2019 (Hotz and Salvo, 2022). For most of 2020 and 2021, the efforts of the Census Bureau were concentrated on optimizing and tuning the parameters of the TDA to make improvements in the accuracy of the PL 94-171 redistricting file. Demonstration files with the updated TDA that included incremental improvements in data accuracy due to changes in the privacy loss budget were also released. The most significant improvement in the new DAS took place in Spring 2021 as a result of a big increase in the privacy-loss budget, and this was eventually adopted by the Bureau's Data Stewardship Executive Policy (DSEP) committee for the 2020 Census PL 94-171 redistricting file, and published on their website in August, 2021.^4^

### 5.2 Uncertain and disputed decisions [2022 onwards]

Even though the finalized DAS was a substantive improvement over the initial versions of the TDA, there continues to be disagreement over DP's impact on the PL 94-171 redistricting data and, thus, the creation of districts that represent minority voters. For instance, in the examination of the final 2010 demonstration data which was produced using the finalized TDA, researchers have found evidence of biases in the drawing and simulations of voting districts (Kenny et al., 2021). These biases primarily arise because the accuracy target of the finalized TDA is maintained at geographies other than at the level of voting districts, such as census blocks.^5^ However, there is a lack of consensus on this matter because others find that the new DAS does not threaten the ability to produce districts with tolerable population balance or to detect signals of racial polarization for Voting Rights Act enforcement (Cohen et al., 2021).

While the final redistricting file with the new DAS was released for public use in August 2021, debate and discussion surrounding other Census products persisted. Of particular concern has been the content of the Demographic and Housing Characteristics (DHC) product which contains substantial more detail than the PL 94-171 redistricting file.^6^ Given the wider variation of demographic and household characteristics available in the DHC, the data is often used to study social phenomena over smaller geographic areas. As a result, social scientists have been concerned about the accuracy of the data in the case of such fine-grained analysis. For instance, a strong critique posed by social scientists against the 2020 DAS is based on the distortions it introduces for group-specific mortality rates, especially minority racial and ethnic groups (Santos-Lozada et al., 2020; Hauer and Santos-Lozada, 2021). However, other researchers have found no evidence of distortions in premature mortality rates calculated using DP-infused data (Krieger et al., 2021). In addition, it is important to note that all these studies based on mortality rates use an earlier version of the 2010 PL 94-171 redistricting demonstration data with a smaller privacy-loss budget since the earlier files contain information about age groups which was absent in the final version of the data that had a larger privacy-loss budget.^7^ As a result, until 2022, researchers were unable to evaluate the impact of the larger privacy-loss budget on the accuracy of mortality rates and other analyses based on granular, individual level data.

In 2022, first March and then August, demonstration products that applied the TDA to 2010 DHC data were released to the public. The demonstration products provided users with the opportunity to provide feedback to help further inform the development of the DAS. For each demonstration data product, the public was provided a minimum of 30 days to review and provide comments. A review of the user comments provided for the second demonstration product indicates that accuracy of the data, particularly accuracy for small populations and/or small geographies, continued to be the primary concern amongst users of the DHC (Devine and Krause, 2022). Based on this feedback, the privacy-loss budget and other parameters were adjusted and then finalized by the DSEP for the production of the 2020 Census DHC that was released in May 2023. The final Detailed DHC File A (Detailed DHC-A) providing more detailed information on 370 racial and ethnic groups, and 1,200 American Indian and Alaska Native tribe and village population groups, was also released in September of the same year.

Due to improved race and ethnicity questions, coding, and processing in the 2020 Census, the Detailed DHC-A provides population counts for more detailed racial and ethnic groups than any previous Census. Since many of these detailed groups have relatively small population numbers, publishing statistics for them while maintaining confidentiality can be a challenge. As a result, and in response to user feedback, the Bureau adopted an adaptive design—“a data-driven framework for choosing which statistics to publish”—in their DAS for the Detailed DHC-A (Bureau and Team, 2023). The algorithm underlying the new DAS was named SafeTab-P. The framework works by adjusting the amount of age data published for a population group based on a combination of predetermined criteria and the level of geography. As a result, the Census Bureau is able to ensure confidentiality by varying the amount of detail provided for each racial and ethnic group based on their population count. In fact, total population counts for groups with a national population smaller than 50 in the 2010 Census are only made available at the national and state levels. The type of tables generated for the other larger detailed groups are determined by SafeTab-P.

Similar to the DHC, a Proof of Concept for the Detailed DHC-A was released on January 31, 2023 for a 30-day public comment period that ended March 2, 2023. The Proof of Concept outlined how SafeTab-P uses an adaptive design to ensure confidentiality and the implications of that on the data available for different racial and ethnic groups. Majority of the feedback received by the Bureau emphasized the need to make the Detailed DHC-A easier for data users to understand and use. The possibility of data users not paying heed to the Census Bureau's caution against aggregating Detailed DHC-A data was of particular concern to these commentators (Bureau, 2023b). The final Detailed DHC-A product was released on September 2023. In response to the user feedback provided on the Proof of Concept, the Bureau also released an expanded guidance on using the Detailed DHC-A as part of the final technical documentation. They have also indicated that guidance materials would continue to be published in the future.

At the start of 2021, the Bureau also found themselves embroiled in a controversy over the Privacy-Protected Microdata Files (PPMF) released by them.^8^ Noisy Measurement Files (NMFs) are a direct output of applying the TDA to the Census Edited File (CEF). Since NMFs do not undergo any post-processing, they are quite hard to interpret because of issues like negative and non-integer values for population counts. From a theoretical standpoint, releasing the original NMF itself would not have compromised respondents' privacy. However, the Bureau released the PPMF of the Redistricting data instead to ensure that the final tabulated statistics met data consistency requirements (such as non-negative and integral values for population counts). This post-processing decision created substantial discontent among Census data users and privacy experts alike (Dwork et al., 2021b; McCartan et al., 2023) as it increased the possibility of introducing biases in the released statistics. More significantly, not only were these biases challenging to correct, but future policy decisions based on these data were found to have significantly harmful effects. The public discourse that followed Dwork et al. (2021b), Schneider (2021a,b) included, amongst many things, a court case (v. Census Bureau, 2022) in which the plaintiff sued the Census Bureau in October 2022 under the Freedom of Information Act (FOIA). The plaintiff's assertion was that the Bureau had failed to provide a timely decision on his request for obtaining the NMFs. Although the Census Bureau initially denied the FOIA request to release the NMFs in December 2022, they later reversed course and announced that they would release them in January 2023. Finally, in April 2023, the Bureau released a demonstration NMF for the 2010 Redistricting Data, followed by an NMF for the 2010 DHC in June 2023 (Bureau, 2023a).

In June 2022, CNSTAT convened another workshop (National Academies of Sciences, Engineering) to discuss and solicit feedback from the data user community on the DHC demonstration files produced by applying the TDA on the 2010 DHC data. Similar to the previous workshops, a wide variety of Census data users, such as demographers, academics, and local, state, and federal government officials, presented their analyses at this workshop. During the discussions, it was acknowledged that improvements in the TDA and post-processing constraints had led to improvements in data accuracy. However, the need for better post-processing steps and greater transparency about them were also highlighted. In particular, a public request was made to release the NMFs or an approximation of them so that users could evaluate and better understand that part of the process. While most of the sessions during the two-day workshop were dedicated to evaluations of the DHC demonstration for a variety of different use cases, the final group of sessions was dedicated to discussions on how the Bureau can obtain and evaluate feedback on its data products. The importance of communication, especially the education of users, was explicitly emphasized by the speakers.

### 5.3 How effective was the Census Bureau's communication strategy?

There are clear successes in the Census Bureau's overall communication strategy about their DP-based DAS that can be replicated by other organizations looking to incorporate DP into their own privacy systems. The recognition of the importance of soliciting diverse user perspectives for different use cases lies at the center of the Bureau's success in communicating about the DP framework for the 2020 Census. The different approaches adopted by the Bureau to collect inputs from their user community and the Bureau's efforts to respond to user concerns and feedback has played an important role in ensuring a wider acceptance of the new DP-based DAS today in comparison to 2019, when the first demonstration product was released. The Bureau has also benefited from communicating and engaging with their user community as is reflected in the significant improvements made to the TDA till date, especially in ensuring the continued utility of the decennial Census data for different use cases.

Of particular importance has been the use of demonstration products by the Bureau to communicate about and receive feedback on the operation of their DP-based DAS. These demonstration products have functioned as a common thread for researchers and policymakers of different backgrounds to evaluate and discuss the effects of different versions of the TDA (and SafeTab-P) on the Census data; with each other and the Census Bureau. It is also likely that these products represent different things for different stakeholders involved in the process. For instance, the demonstration products represent for the Bureau, amongst many other things, a way to communicate about a new system that helps them effectively perform one of their main duties of maintaining the confidentiality of Census data. However, for researchers familiar with DP but not using Census data for their own research, the demonstration products represent a compelling case for large scale deployment of DP which lends itself to more fine-grained DP discussions on topics such as choosing a proper privacy budget. In contrast, researchers and practitioners unfamiliar with DP but who regularly work with Census data are more likely to rely on the demonstration products as means to ascertain how a DP-based DAS biases the study of social phenomenon. In the absence of the demonstration products or another object that could have served as a common thread, it would have been significantly harder for the different user groups to discuss the impact of the TDA with each other, especially if they were applying the same DP criterion to different datasets. Thus, drawing on the ISC framework, we argue that the demonstration products operate as the critical boundary objects required to ensure that the scientific communication about differential privacy is inclusive.

However, the presence of boundary objects alone does not ensure that the Census Bureau followed a truly inclusive communication strategy. It is also important to determine the extent to which the three ISC traits of intentionality, reciprocity, and reflexivity are concurrently reflected in the Bureau's communication. As traced through the case study in this paper, we find evidence of a constantly evolving communication strategy that did not explicitly embrace inclusivity at the start but has grown over time to acknowledge the importance of ensuring inclusive communication with the diverse users of the decennial Census data and taken steps toward achieving it.

The Census Bureau released the first Federal Register Notice in 2018 to collect feedback from its users about the proposed new DP-based DAS. However, the Bureau did not provide any further explanation for why this feedback was being sought out. Instead, they only provided a “general statement about improving confidentiality protection” (Hotz and Salvo, 2022, p. 10). As a result, the request generated a great deal of concern and confusion amongst the users. Drawing on the ISC framework, we propose that this approach lacks an intentional consideration of the audience, especially marginalized identities, with whom the Bureau was communicating. There was no consideration of the asymmetries in knowledge pertaining to DP that might exist amongst the users of the decennial Census data which would affect the extent to which different groups could provide feedback on the adoption of DP. This lack of intentionality in the Federal Register Notice also hindered the reciprocity of the process by creating unequal capacities between the user groups to shape the DP adoption process. In addition, by not providing users information about their reasons for wanting to adopt the new DP-based DAS, the Bureau also created an unequal relationship between themselves and the Census data users. They placed themselves in a position of power as a knowledge producer and relegated the Census data users to the status of uninformed consumers. Although the Federal Register Notice was severely lacking in ISC traits, we recognize that the Bureau's decision to solicit feedback was nevertheless a step in the right direction and demonstrates an inclination toward establishing reciprocity through the co-creation of knowledge that draws on varied forms of expertise.

The 2019 and 2022 CNSTAT workshops that were conducted after the release of the demonstration data lend themselves better to the three traits of the ISC framework. The discussions during these workshops and the subsequent actions they engendered demonstrate how inclusivity has become a central feature of the Bureau's communication strategy over time. Below we identify and elaborate upon three key characteristics of the communication before, during, and after the CNSTAT workshops that reflect the ISC traits.

#### 5.3.1 Data-user awareness

The first workshop in 2019 was conducted after the Census Bureau had formally announced its intention to adopt a DP-based DAS. In fact, prior to every workshop, the participants were always informed about the specific purpose for which their feedback was being sought, thus creating a more equitable relationship between the Bureau and the Census data users based on reciprocity. In keeping with the trait of intentionality, the Bureau also made a concerted effort during each workshop to share the rationale behind their decision-making. Both CNSTAT workshops started with panel discussions led by the Bureau leadership where they provided insight into the factors motivating the Bureau's decisions and addressed questions from participants. For instance, at the start of the 2022 workshop, the Bureau leadership emphasized the importance of data equity and communication strategies as the implementation of the DP-based DAS is expanded in the future. Furthermore, at the conclusion of each workshop, Bureau representatives summarized what they had learned from the workshop proceedings and their intended plan of action moving forward - a critical demonstration of reflexivity. In fact, the proceedings for both workshops have been documented in detail and made publicly available which adds another layer of accountability that the Bureau has adopted for itself. Thus, we find (a) intentional engagement by the Bureau to communicate the science behind their decision-making; (b) reciprocity in their efforts to establish an equal relationship between the Bureau and the data users in knowledge sharing and knowledge production; and (c) reflexivity in their future action plans formulated based on the workshop proceedings. In other words, we see traits of intentionality, reciprocity, and reflexivity exhibited in how the Bureau communicated with all its users during and after the CNSTAT workshops.

#### 5.3.2 Diversity of user opinions

Another facet of the CNSTAT workshops that resonates with the ISC traits of intentionality and reciprocity is the diversity of opinions encouraged and expressed in the sessions that constituted each workshop. In 2019 and 2022, a large share of the workshop proceedings were dedicated to understanding how the new DAS affects different use cases of the decennial Census data. Thus, the focus of each session was on different groups of data users and their needs - drawing researchers from disciplines as varied as computer science and sociology, along with advocacy organizations. Such an approach speaks directly to the reciprocity trait that calls for recognizing the value of varied forms of expertise and the intentionality trait that encourages intentional engagement with people representing a diverse group of identities.

We also find evidence of ISC's reflexivity trait in the diversity of user opinions that constituted the CNSTAT workshops. An extended focus on different uses cases can often result in bringing data accuracy concerns to the forefront and pushing other topics like privacy to the background. The Bureau was able to reflect on and recognize the possibility of such inequities arising in the communication by different user groups during the workshops. Thus, to ensure that all voices are heard, time was intentionally allocated during both workshops for presentations and discussions focused on issues and concerns pertaining to privacy that influence the development of a DP-based DAS. For instance, a panel discussion with 5 privacy researchers was conducted during the 2019 workshop, which included danah boyd (Microsoft Research), Omer Tene (International Association of Privacy Professionals), Helen Nissenbaum (Cornell Tech), Paul Ohm (Georgetown Law Center), and Daniel Barth-Jones (Mailman School of Public Health, Columbia University). It is also important to note that while the first workshop was in-person, the second one was conducted in a hybrid format to encourage more widespread attendance. Thus, we see the Bureau's ability to systematically reflect on the diverse needs of its users and adapt to address the needs of users who might not have had the resources to attend the workshop in person. Such an approach also demonstrates intentionality in trying to engage with users who might not have been able to attend the first workshop in-person.

#### 5.3.3 Information accessibility

Lastly, a critical component of the Census Bureau's communication strategy that resonates both with intentionality and reciprocity has been the wide ranging access to information on the DP-based DAS that the Bureau has made publicly available. Both CNSTAT workshops were streamed live so that people unable to attend them in-person at Washington DC could listen to the sessions virtually. In addition, videos and slides of all the sessions conducted at each workshop can be accessed, at no cost, through the CNSTAT website or the National Academies website.^9^,^10^ On the completion of each workshop, the Bureau also published a detailed report of the workshop proceedings wherein a summary of all the presentations in each session is documented, along with a summary of the discussions that took place at the end of each session (National Academies of Sciences, Engineering; National Academies of Sciences and Medicine, 2020). These reports are extremely thorough and even document the questions asked by the participants and the responses provided by the presenters and panelists. In fact, similar to the other DP content generated by the Bureau, the reports for the conference proceedings can be downloaded for free from the Bureau's website which makes them easily accessible to the general public. Overall, all these strategies to disseminate information reflect intentionality on part of the Bureau to engage with as wide an audience of data users as possible (irrespective of their resource constraints) and establish transparent and equitable relationships with them through knowledge sharing.

Moving beyond the CNSTAT workshops, we also find evidence of ISC traits reflected in other mechanisms used by the Bureau to communicate about their DP-based DAS at different stages. The Bureau has not only ensured that the workshop proceedings are easily accessible but also attempted to maintain a similar vein of accessibility with respect to its decision-making about the Census data products. On its website, the Bureau has provided a wide variety of information for each data product it has released till date.^11^ In keeping with the theme of intentionality and reciprocity, all the data files necessary for users (PPMFs, MDFs, and now NMFs) are available on the website. In addition, the Bureau also shares a trove of additional information on their website which includes (a) all Census Bureau newsletters associated with a given data product; (b) fact sheets; (c) product development timelines; and (d) summaries of the user feedback provided to the Bureau for each round of product development. Thus, all groups of data users are able to stay updated on the latest developments in the DP-based DAS and use the information to better understand the motivations driving the Bureau's decisions.

In addition to facilitating engagement with diverse user groups, the public availability of all this information has also bolstered accountability by allowing data users to track and ascertain the extent to which the Bureau is paying heed to the feedback provided to them. The Bureau's systematic documentation of the feedback provided to them, allowing users to hold them accountable by making the feedback publicly available, and adapting their approach to reflect this feedback all resonate strongly with the ISC trait of reflexivity. For instance, in the 2022 workshop proceedings, one of the suggestions provided to the Census Bureau during the session on “Observations on Use Cases and Needs” was that the user feedback provided to the Bureau should be cataloged and be more transparent. It was also proposed that the Bureau should communicate “when data products will be released based on the feedback and the needs users have already expressed” (National Academies of Sciences, Engineering, p. 100). At the time of writing this paper in April 2024, and in keeping with the feedback, the Census Bureau's website provides (a) a summary of the user feedback received by them for each product at every stage, and (b) detailed timelines about product development and releases.

Overall, we find clear evidence that the Bureau's communication strategies concurrently resonate with all three traits of ISC and employ boundary objects, and have thus helped establish equitable relationships between (a) the producers of knowledge (the Bureau) and its consumers (Census data users), and (b) different types of users. However, it is also essential to recognize that ensuring inclusivity is an ongoing process and that there continue to be many areas in which the Bureau can further improve its communication strategies to resonate more strongly with the ISC framework. One such case in point is the dispute over the NMFs, which led to an open letter (Dwork et al., 2021a) and a FOIA lawsuit being filed against the Bureau (v. Census Bureau, 2022). We suggest that these events demonstrate a gap in the Bureau's engagement with its stakeholders due to a lack of intentionality and reciprocity. Not only was the Bureau unable to identify and address their users' needs for the NMF data files to undertake more granular analyses, but they also chose not to communicate at the outset the reasons for not releasing these files. Recently, Abowd (2024) has clarified that the Bureau initially denied the FOIA request because the NMFs at the time were not designed for direct publication. The initial storage format of the NMFs mixed confidential information with noisy measurements. It was only after new software was written to extract the noisy measurements from the confidential information that the NMFs could be publicly released. Although the Bureau's actions are justified in hindsight, the need for NMFs to be further processed prior to public dissemination (to the best of our knowledge) was not communicated to users at any time prior to the filing of the FOIA lawsuit. It is possible that the need for a lawsuit would not have arisen if, from the start, the Bureau had shared reasons for their hesitancy toward releasing the NMFs in their original format. An important point to note here is that the granular analyses facilitated by the NMFs are critical for the study of social inequities (Dwork et al., 2021b). Thus, for a period of time, the absence of inclusive communication possibly hindered the redressal of social inequities in the real world. Additionally, the resources directed toward the FOIA lawsuit could have been used elsewhere if the Bureau had engaged with users more intentionally to facilitate reciprocity and reflected on the consequences of their decision to withhold information.

The events around the FOIA lawsuit are one example of inequitable communication that occurred during the development and implementation of the DP-based DAS. The emergence of such inequities is expected to a certain extent, given that the Bureau occupies a position of power and authority as the primary producer of all information pertaining to the new DAS and has sole discretion on how much information to share. However, it is also for this reason that it is integral to hold the Bureau accountable for following inclusive communication practices. Otherwise, as we saw in the aforementioned FOIA example, there exist real possibilities for the use of DP to impeded the study and redressal of social inequities. A matter of particular concern has been the failure of the Bureau to communicate how uncertainty has always been present in the past decennial Census data products that employed earlier versions of the DAS. As Abowd and Hawes (2023) point out, the Bureau has done a poor job of communicating about the role of noise injection in all the decennial Census prior to 2020 and this has caused more confusion during the transition to the DP-based DAS. In fact, many Census data users believe that no biases exist in the data that use earlier DAS like swapping (Dwork et al., 2021b) because statistical uncertainty within non-DP DAS has previously never been quantified and publicly discussed. Thus, there exists a pressing need for the Bureau to develop mechanisms to effectively communicate about statistical uncertainty with users of the decennial Census data, especially how it pertains to swapping. In fact, the importance of the issue was even emphasized during the 2022 CNSTAT workshop in a panel discussion on the way forward for the Bureau in their implementation of the DP-based DAS (National Academies of Sciences, Engineering; Boyd and Sarathy, 2022).

The issues pertaining to the NMFs and statistical uncertainty highlight the additional work that remains to be done by the Bureau to intentionally communicate about the "science of DP" performed by them. Nevertheless, based on the 2022 workshop proceedings, it appears that the data user community largely appreciates the Bureau's efforts to engage as many diverse use cases of the Census data as possible. However, data users have also identified areas where the Bureau's communication and engagement has been insufficient. Workshop participants especially highlighted the need for the Bureau to engage with local communities so that they are able to understand “why it is that they are not able to access what they feel they need to make the correct community decisions and policy” (National Academies of Sciences, Engineering, p. 97). These conversations are particularly important for small groups, especially those who lie at the intersection of multiple vulnerabilities, since their data might not be publicly available for analysis due to the noise infusion by the new DAS.^12^ A recourse proposed for this problem is to connect these small groups to others who can do the required analysis for them rather than creating disengagement by prohibiting any form of granular analyses. Once again, this suggestion from the data users highlights the importance of inclusive communication in ensuring that the equity considerations for all groups, particularly marginalized groups, are recognized and addressed during the deployment of a DP-based system.

## 6 Discussion

In this paper, we highlight the importance of ensuring inclusive communication in every stage of the DP life cycle and thus extend the discussion of equitable privacy to define Equitable DP as the design, communication, and implementation of DP algorithms that ensure equitable outcomes. We have outlined how the U.S. Census Bureau has consistently exhibited inclusive communication practices that align with our conceptualization of Equitable DP and also drawn attention to areas where there remains room for improvement. As Abowd and Hawes (2023) have stated, “Perhaps one of the greatest lessons learned from the Census Bureau's experience transitioning to DP is the active role data users can and should play in shaping the disclosure limitation system.” By identifying communication practices that have facilitated the active role of a diverse group of data users, we hope to provide suggestions that can be replicated across organizations to ensure that DP is implemented equitably. More broadly, we also expect that increased access to DP knowledge through better communication practices will contribute to increased public trust in DP based practices (Cummings et al., 2021).

An important limitation of our study is that we do not evaluate the other components of Equitable DP with respect to the Census' DP-based DAS, i.e., we do not evaluate whether the design and implementation of the DP-based DAS created data distortions that had repercussions for equity. Hence, we cannot make conclusive statements about the extent to which the new DAS adheres to our definition of Equitable DP. However, it is important to recall that an exhaustive evaluation of the Census' DAS is not the main focus of our paper. Instead, we use the Census Bureau as a case study to demonstrate the importance of ensuring inclusive communication during the deployment of DP algorithms, and thus propose that it should be included in the conceptualization of Equitable DP.

While discussing how the Bureau's Equitable DP practices pertaining to communication can be replicated across different organizations, we must be cognizant that inclusive communication is most likely to be realized differently in government agencies vs. private organizations. Our case study of the Bureau and their use of DP best serves as a guide for other government agencies looking to adopt the Equitable DP approach because of the similarities in responsibility and accountability expected of government institutions. Since government institutions are subject to more accountability (to other government institutions and the general public), they are more likely to voluntarily adopt inclusive communication practices to engage with their users so as to meet the levels of accountability and transparency expected of them. In comparison, private organizations are primarily accountable to investors and operate on deadlines determined by profit margins. Thus, it is less likely that the communication dimension of Equitable DP will be realized in these organizations through the same types and levels of user engagement as can been seen in the case of government agencies.

Variations in inclusive communication practices imply that the type of boundary objects will also vary across government and private organizations, and in some cases be completely absent. For instance, demonstration products are critically important boundary objects for the Census Bureau since one of their key goals is to solicit feedback from diverse users and facilitate discussion between these groups. However, an organization not motivated to soliciting feedback and responding to it is also unlikely to develop demonstration products or other objects that facilitate communication across diverse groups. We know that many private organizations are not intrinsically motivated to seek user feedback from diverse groups due to a lack of accountability. Consequently, even though private organizations might choose to communicate their DP related decisions to users, they are less likely to feel motivated to actively seek inputs from different user groups on these decisions and then respond to user inputs, especially for groups that constitute a minority of the user population. The absence of intrinsic motivation to develop a boundary object in turn will prevent private organizations from adopting inclusive communication practices. Instead, we propose that upholding an Equitable DP framework in private organizations is more dependent on the development of regulations that hold these organizations accountable for justifying their choices in the design and implementation of their DP systems (Gillis and Simons, 2019).

In addition to the type of organization, inclusive communication practices inherent to Equitable DP should also be tailored to the geography and size of the data user community. For instance, since the users of the decennial Census data are dispersed across the entire country, the Bureau has relied heavily on various online communication mechanisms such as webinars, blogs, and newsletters for their outreach and for soliciting user feedback. Further, these feedback periods have typically been conducted only 2-3 times for each product, and a widespread user community has also possibly limited the number of in-person workshops that can be conducted, even though they are the driving force of the inclusive communication strategy employed by the Bureau. After all, the resources required to conduct such workshops increase as the number of participants increases. A case in point is that the 2022 workshop was attended (in person and virtually) by over 1,000 people, and yet one of the key workshop takeaways was that many users of the DHC continue to be unidentified and need to be brought into the decision-making fold by the Bureau (National Academies of Sciences, Engineering).

The data user community will likely be smaller and less dispersed for government agencies at the state or county level looking to adopt an Equitable DP framework to preserve the privacy of their local datasets. As a result, the nature of engagement possible with these communities is also different, with the use of participatory methods of engagement being more feasible. While webinars, blogs, and newsletters are still highly effective tools to ensure timely communication, conducting frequent and in-person feedback sessions with smaller user communities is easier and less resource-intensive. Thus, resources permitting, local agencies can conduct annual or semi-annual workshops where diverse data users can share their feedback and engage in participatory decision-making. Additionally, user input can be sought to determine how often and in what format they would like to provide feedback to their local government agency so as to prevent users from feeling burnout from providing feedback, an issue that was highlighted during the 2022 workshop. Local agencies can also experiment with other novel user engagement strategies, such as using media (television, radio, print, etc.) to reach the unidentified users of their datasets and conducting training workshops to equip vulnerable communities with the skills required to analyze their own data.

Ensuring equitable outcomes in the design and implementation of DP is a growing field and has a gamut of intricacies that are being resolved on a daily basis. The addition of inclusive communication in this conversation is essential but also further complicates the answer to what a privacy, accuracy, and equity-preserving DP framework looks like. In this paper, we do not attempt to provide a singular answer to this question. Instead, we highlight specific practices that the U.S. Census Bureau has demonstrated to be effective in working toward the establishment of Equitable DP. While we recognize the contextual nature of these practices, we propose that they provide worthwhile insights that can be adopted by other organizations looking to adopt an Equitable DP approach. We anticipate that the suggestions we have have put forth in this paper will be particularly efficacious for other statistical agencies in the US looking to modernize their equivalent of the Census Bureau's DAS.

## Data availability statement

The original contributions presented in the study are included in the article/Supplementary material, further inquiries can be directed to the corresponding author.

## Author contributions

VK: Conceptualization, Formal analysis, Investigation, Methodology, Writing – original draft, Writing – review & editing. TM: Conceptualization, Formal analysis, Investigation, Methodology, Writing – original draft, Writing – review & editing.

## Footnotes

^1^The privacy accuracy trade-off is not limited to DP and applies to all privacy preserving mechanisms. DP's unique feature is that it allows for the numerical quantification of the trade-off whereas other privacy preserving mechanisms do not permit the same (Wood et al., 2018)

^2^For an illustrative example of how the amount of skew is more pronounced for smaller populations, please refer to Table 2 in the 2020 Census Brief on “Disclosure Avoidance Methods for the Detailed Demographic and Housing Characteristics File A (Detailed DHC-A): How SafeTab-P Work” (Bureau and Team, 2023)

^3^The term “statistical data privacy” (SDP) captures multiple frameworks that tackle the challenges of sharing statistical information while maintaining the privacy of entities in the data. SDP includes but is not limited to DP

^4^The Bureau was able to optimize the DAS to create an accuracy target to ensure that the largest racial or ethnic group in any geographical entity with a total population of at least 500 people is accurate to within 5 percentage points of the 2010 published counts at least 95 percent of the time (Bureau, 2021) In addition, the Bureau was also able to improve accuracy in different legal, administrative, and political geographic areas, which had been a problem in the earlier demonstration products (Hotz and Salvo, 2022).

^5^Each voting district typically consists of several census blocks

^6^The DHC data includes information about the American population based on 5-year age groups, sex, race, Hispanic or Latino origin, household type, relationship to householder, group quarters population, housing occupancy, and housing tenure.

^7^The final version of the PL 94-171 redistricting demonstration files that use the new TDA with a larger privacy budget do not contain detailed information on age. Instead, data on age was only released in two broad categories: under 18 and 18 and over (Hotz and Salvo, 2022).

^8^For an explanation on the different processing stages that confidential Census data undergoes and the files generated at each stage, refer to Appendix 1.

^9^Materials for the December 2019 workshop are available at: https://www.nationalacademies.org/event/12-11-2019/workshop-on-2020-census-data-products-data-needs-and-privacy-considerations.

^10^Materials for the June 20233 workshope are available at: https://www.nationalacademies.org/event/06-21-2022/2020-census-data-products-workshop-on-the-demographic-and-housing-characteristics-files.

^11^ https://www.census.gov/programs-surveys/decennial-census/decade/2020/planning-management/process/disclosure-avoidance/2020-das-development.html

^12^For instance, under SafeTab-P, detailed groups with a national population smaller than 50 in the 2010 Census are preset to receive total population counts at the nation and state levels only (Bureau and Team, 2023). Thus, data for more granular analyses is not available for these groups.
