# Supplementary material for: Equitable differential privacy
Source: Front Big Data. 2024 Aug 16;7:1420344. doi: 10.3389/fdata.2024.1420344 (PMC11363707; doi:10.3389/fdata.2024.1420344)
Supplement: Supplementary file 1 [file Data_Sheet_1.pdf]

1 BACKGROUND ON DIFFERENTIAL PRIVACY

Since the introduction of differential privacy, there has been an explosion in the research regarding the theory and implementation of this technology (Vadhan, 2017; Machanavajjhala et al., 2017; Garrido et al., 2022). For a more in-depth discussion on the consequences of using DP from a social science perspective, we refer to (Oberski and Kreuter, 2020). For more technical details regarding DP, including its formal mathematical definition, we refer to (Dwork and Roth, 2014).

As the use of DP systems becomes more widespread, it has become increasingly important to understand how non-experts and end users perceive DP and how knowledge about DP can be disseminated to them most effectively. Bullek et al. (2017); Xiong et al. (2020); Karegar et al. (2022) study how helping end users understand DP affects their willingness to share information and sets their expectations for privacy guaranteed by DP systems. Bullek et al. (2017) find that showing end users the amount of noise added to make their responses private increases their willingness to share information. On the other hand, Xiong et al. (2020) find that explaining the implications of a privacy-preserving technique vs. the technique itself increases users' comprehension and willingness to share their information. Karegar et al. (2022) evaluate the usefulness of metaphoric explanations for DP and find that although metaphors are helpful in conveying that noise perturbation can protect privacy and that there is a utility tradeoff, very high-level explanations can also lead to incorrect privacy expectations and lower users' willingness to share. Xiong et al. (2022); Wen et al. (2023) use descriptive illustrations to help end users understand more complex models of DP and find that illustrations can be an effective method in communicating DP. Cummings et al. (2021) extend this line of work to examine whether the privacy concerns of end users align with the privacy protections offered by DP. Their results suggest that users are more likely to share their information when they can understand the nature of information leaks that DP protects against and when such leaks are less likely to happen.

2 BACKGROUND ON DISCLOSURE AVOIDANCE SYSTEMS USED BY THE U.S. CENSUS BUREAU

The Census Act requires the U.S. Census Bureau to protect respondent confidentiality at every stage of the data life cycle. The main intention of these confidentiality provisions is to ensure that a specific individual, household, or business cannot be identified from the statistics and tabulations that are made publicly available by the Bureau. Information collected about respondents' names, date of births, addresses, and telephone numbers are never released publicly. In contrast, information about respondents' race, age, sex, etc. are aggregated and published but are protected by disclosure avoidance methods to protect every individual respondent's identity.

Disclosure avoidance systems (DAS) help de-identify personally identifiable data to prevent a statistic from being traced back to a specific respondent. The Census Bureau has been using different types of DAS since the 1930s. Their first system entailed not publishing certain tables from small geographic areas to prevent indirect disclosure, the scope of which was expanded in the 1970s and 1980s. In the 1990s, the Census Bureau started using more sophisticated DAS such as data swapping to inject noise into the data that is collected. Under this method, the geographic identifiers of certain households are swapped with the identifiers from nearby households with similar characteristics to prevent disclosure. To ensure that no personally identifiable information is disclosed, information about the specific details of swapping is not released either. In the subsequent 2000 and 2010 Censuses, the Bureau continued to use data swapping but

also adopted other techniques to address new internet threats. These techniques included top- and bottom-coding (grouping values above or below certain thresholds into broader categories), blank-and-impute techniques (replacing actual responses with statistically generated data), and table and cell suppression (not publishing certain data points). Even though some amount of loss of confidentiality is inevitable for any publicly available dataset (Dinur and Nissim, 2003), the fact that these DAS did not provide a formal mechanism for measuring such privacy loss was sparingly acknowledged during these transitions (Hawes, 2020).

The absence of any discussion on confidentiality loss, however, eventually became a key point of contention when the Census Bureau adopted the new framework of differential privacy (DP) in 2020 to further modernize their DAS. The decision to adopt a new DAS was motivated by the belief that the existing DAS were too fragile to ensure the continued confidentiality of the granular data collected by the Census. Many voices within the Bureau felt that the rapid progress in computing power and the exponential growth in the amount of individual level data independently held by commercial aggregators like Facebook, X, LinkedIn, Apple, Microsoft, etc. has made re-identification increasingly easy (Abowd et al., 2022). The reconstruction of Census records by external parties has always been a serious concern for the Bureau because it makes it easier for bad actors to target people, particularly those from marginalized communities, for fraud, disinformation and other harms. Additionally, it also undermines the public's trust in the confidentiality of their Census responses (Nanayakkara and Hullman, 2023). As a result, the Bureau has had a longstanding practice of undertaking its own reconstruction attacks to assess the efficacy of their DAS. For instance, after the 2010 Census, the Bureau simulated an internal reconstruction attack on the 2010 Census data based on published tables on race, ethnicity, sex, and age from the 2010 Census (Abowd et al., 2023). The new DAS based on the DP framework was primarily adopted because it made it difficult for an outside party to reconstruct individual records and re-identify respondents based on aggregated Census data that is publicly released. At the same time, since DP offers a formal way to account for privacy loss and put limits on it, the issue of privacy loss budgets that determine how much accuracy is possible for different levels of privacy (i.e., privacy-accuracy trade-offs) became a topic of intense public debate that continues till date.

Although the Census Bureau framed the adoption of the new DP-based DAS as facilitating the maintenance of respondent confidentiality, many social scientists and policy practitioners have opposed the adoption of the new DAS. The opposition is grounded in the argument that the likelihood and the accuracy of reconstruction attacks is substantially low and the chances of other harms arising from the use of DP are substantially higher (Ruggles et al., 2019). For instance, both sides have varied interpretations of the Census Bureau's ability to accurately reconstruct 46.48 percent of the individual-level confidential records in an internal reconstruction attack based on several published tables on race, ethnicity, sex, and age from the 2010 Census data protected using the older DAS (Abowd 2021). While this 50 percent success rate has been a cause for concern for the Census Bureau and some researchers, others have argued that the potential for privacy loss in the absence of a DP-based DAS is not sufficiently large enough to justify the associated accuracy loss that arises when DP is implemented (Ruggles et al., 2019; Santos-Lozada et al., 2020; Hauer and Santos-Lozada, 2021).

When the Bureau decided to implement the new DP-based DAS, it created demonstration products by applying the proposed DP algorithm to the 2010 Census data. The first demonstration products were created for the P.L. 94-171 Redistricting Data. [13]. These demonstration products were then released to the public

[13] The PL 94-171 redistricting file is the Census data product that is employed for the redistricting process and the U.S. Department of Justice's enforcement of the Voting Rights Act of 1965

to (a) demonstrate that the new DP-based DAS could effectively protect privacy at the scale of the 2020 Census, (b) assure Census data users that the released data would continue to be of the same high quality as earlier, and (c) solicit feedback from them (Abowd and Velkoff, 2020). The users of the decennial Census data employed these demonstration products to illustrate the negative implications of the accuracy loss that arises through implementation of DP. In particular, the barriers that the accuracy loss creates to the study of social phenomena aroused a great deal of concern within the data user community. For instance, researchers demonstrated how estimates of U.S. mortality rates and Covid-19 mortality rates in the U.S. are significantly different when they are estimated using the 2010 demonstration data protected by DP as opposed to the original estimates without the noise (Hauer and Santos-Lozada, 2021; Santos-Lozada et al., 2020). The substantially larger divergences from the original estimates for vulnerable groups and those living in less populous areas was particularly troubling for researchers and policymakers since these patterns often inform policy. In response, the Census Bureau began its engagement in a multi-year process of soliciting feedback from its users, addressing their concerns through modifications in the DP based DAS, and then soliciting more feedback on updated products.

3 PROCESS OF DATA ANONYMIZATION USING TDA AND SAFETAB-P

Before delving into the intricacies of the Bureau's engagement with its users' concerns, it is first important to understand how Census data is processed prior to publication. For each Census product, there exists a Census Edited File (CEF) which is not accessible to the public because it contains confidential Census data that has not been anonymized. When the DAS is applied to the CEF for confidentiality protection, an intermediate file called the Noisy Measurement File (NMF) is created. Since the noise added to records in the NMF can create inconsistencies such as negative and non-integer values for population counts, a post-processing step is applied to remove such inconsistencies and obtain the Microdata Detail File (MDF). Finally, the data from the MDF files is then used to create the easily understandable and usable data tables published by the Bureau in the form of Privacy-Protected Microdata Files (PPMFs).

Note that the process described above is the process of noise-infusion followed by the TDA. In the case of SafeTab-P, the algorithm first creates the tabulations and then noise is added to the tables. For further details, please refer to (Population Reference Bureau and 2020 Census Data Products & Dissemination Team, 2023).
